# Multiple sclerosis with ophthalmologic onset - case report

**Published:** 2018

**Authors:** Dănuţ Costin, Gabriela Mirela Pînzaru, Andra Mădălina Pătraşcu, Anca Moţoc, Andreea Dana Moraru

**Affiliations:** * “Grigore T. Popa” University of Medicine and Pharmacy, Iaşi, Romania; ** Department of Ophthalmology, “Prof. N. Oblu” Emergency Hospital, Iaşi, Romania

**Keywords:** optic neuritis, multiple sclerosis, diplopia

## Abstract

Ophthalmological and neurological signs and symptoms were assessed in a patient diagnosed with retrobulbar optic neuritis associated with multiple sclerosis (MS). The patient presented with progressive decrease of visual acuity, intermittent diplopia, paresthesia of the left arm and equilibrium disturbances. The complete ophthalmologic examination (clinical examination, visual field, optical coherence tomography) along with an MRI exam supported the diagnosis of MS with active lesions associated with retrobulbar optic neuritis. The corticosteroid therapy, followed by betaferon led to the remission of both ophthalmological and neurological signs. The multidisciplinary approach of the case played an important role in the early establishment of the diagnosis as well as the functional recovery of this patient.

## Introduction

Multiple sclerosis (MS) is a chronic inflammatory disease of the central nervous system (CNS), of unknown etiology, in which loss of neurological functions occurs due to an autoimmune demyelination. Multiple sclerosis presents several clinical manifestations associated to brainstem lesions such as impaired motor and sensory functions, cognitive, visual, urogenital, and mental disorders. Periods of remission and exacerbation may alternate [**1**].

Ocular findings in MS include optic neuritis, retinitis, pars planitis, peripheral vasculitis, and ocular motility dysfunction, manifested as nystagmus or diplopia. Optic neuritis, the most common ocular manifestation of multiple sclerosis, may represent the first clinical sign of the disease. Recent long-term follow-up data shows that most patients with demyelinating optic neuritis have an excellent prognosis for recovery of central visual acuity [**2**].

The diagnosis is essentially clinical, although MRI, cerebrospinal fluid measurements, and visual evoked potential studies are often useful for confirmation [**1**].

## Case report

A 30-year-old white male presented with decrease of visual acuity, intermittent diplopia, photophobia in both eyes and paresthesia of left hand. Family medical history was non-contributory.

Ocular and medical history of this patient included: keratoconus in both eyes, operated in the right eye and craniocerebral trauma (at 2 years old) followed by right brachial hemiparesis. In June 2017, after an excessive food and alcohol intake, an acute diarrhea syndrome and vertigo installed. A native CT found no cerebral lesions. After the treatment of the hydroelectrolytic imbalance, the patient was discharged. Fourteen days after this event, he presented in the Ophthalmology Clinic reporting decrease in visual acuity for 5 days, intermittent diplopia, photophobia, and vertigo.

Visual acuity was 0,12 in the right eye and 0,3 in the left eye. Intraocular pressure was 15 mmHg in both eyes. Slit lamp examination revealed segments of intrastromal rings in the right eye and a tight therapeutic contact lens in the left eye. Fundoscopic exam was normal.

**Fig. 1:**
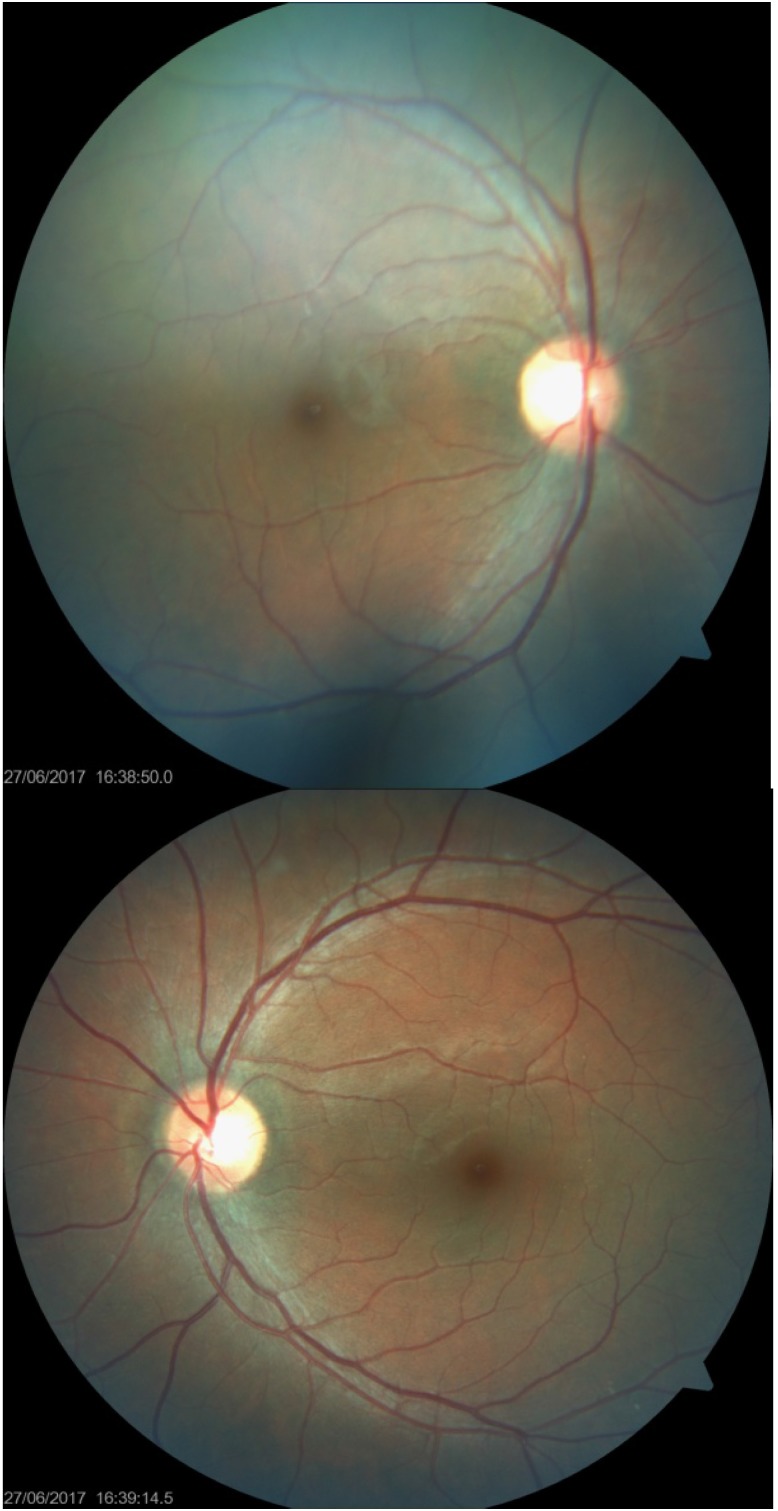
Fundus photography in both eyes

Ocular motility examination revealed impaired adduction and abduction in both eyes, with the maintenance of vertical movements, variable angle (+10-+12 degrees) esophoria at distance and dyschromatopsia in the red-green axis.

The visual field exam showed a slight narrowing in the inferior-nasal sector to 40 degrees from fixation in the right eye and up to 50 degrees from fixation in the inferior-nasal sector in the left eye. 

**Fig. 2:**
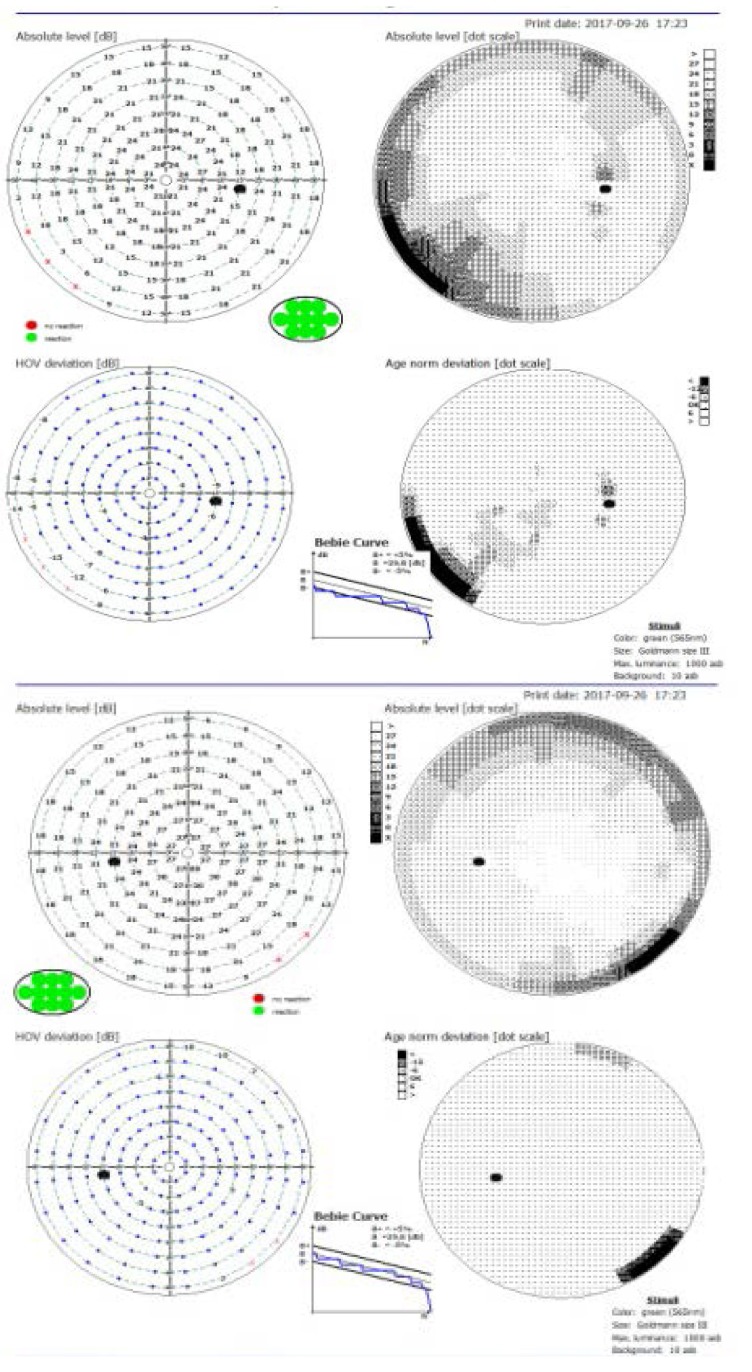
The inferior-nasal visual field defect in both eyes

The optical coherence tomography (OCT) showed reduction of the retinal nerve fiber layer (RNFL) thickness in the inferior sector in both eyes.

**Fig. 3:**
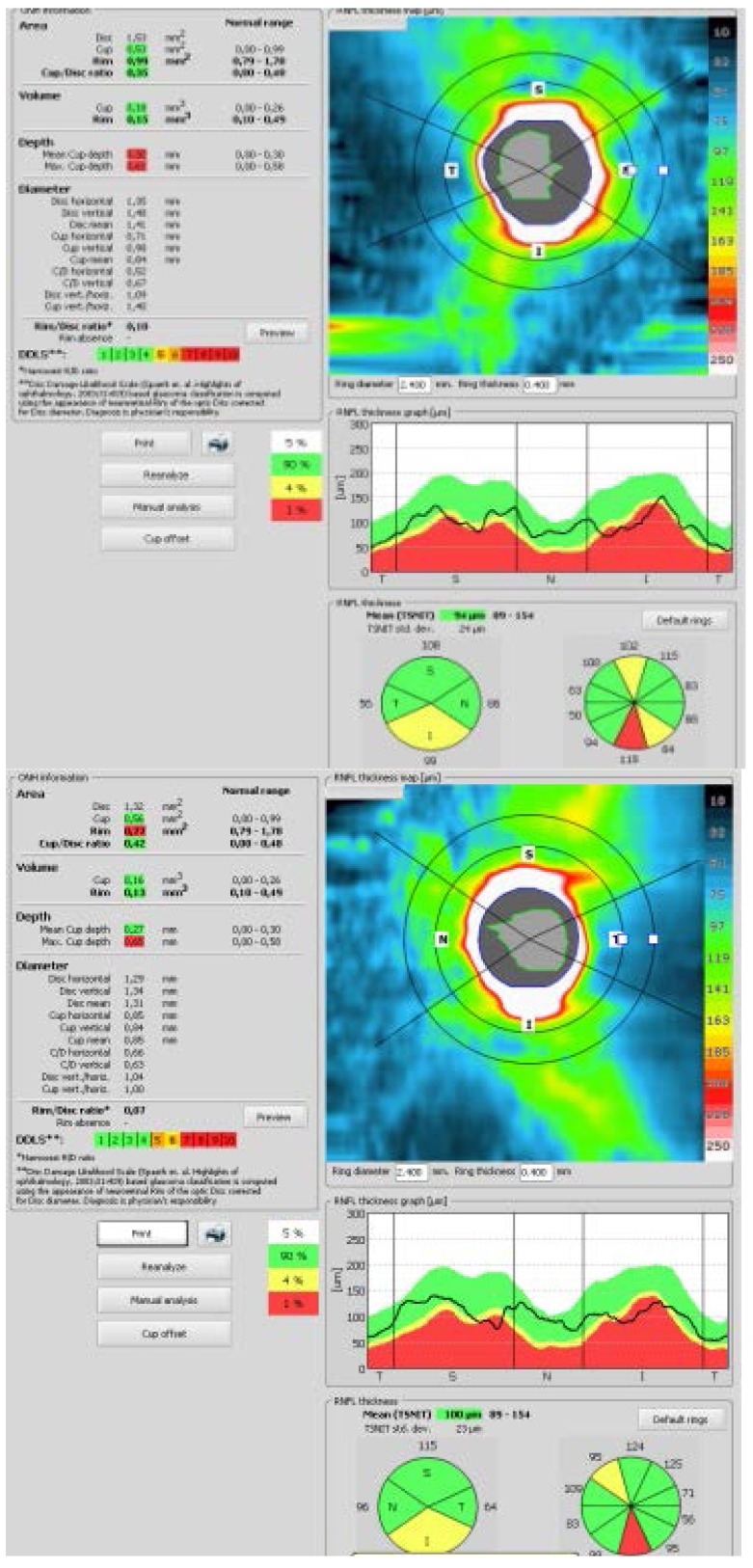
The OCT shows thinning of RNFL in the inferior quadrant in both eyes

Native and contrast cerebral MRI were significant for multiple sclerosis, showing both inactive subtentorial and supratentorial lesions and 4 active, peripheral, gadolinophilic lesions, located posterior median in the pons, in the knee of the right callous body and in the left frontal and occipital lobes. The sequences of angio-MRI revealed no autoimmune vasculitis lesions. 

**Fig. 4:**
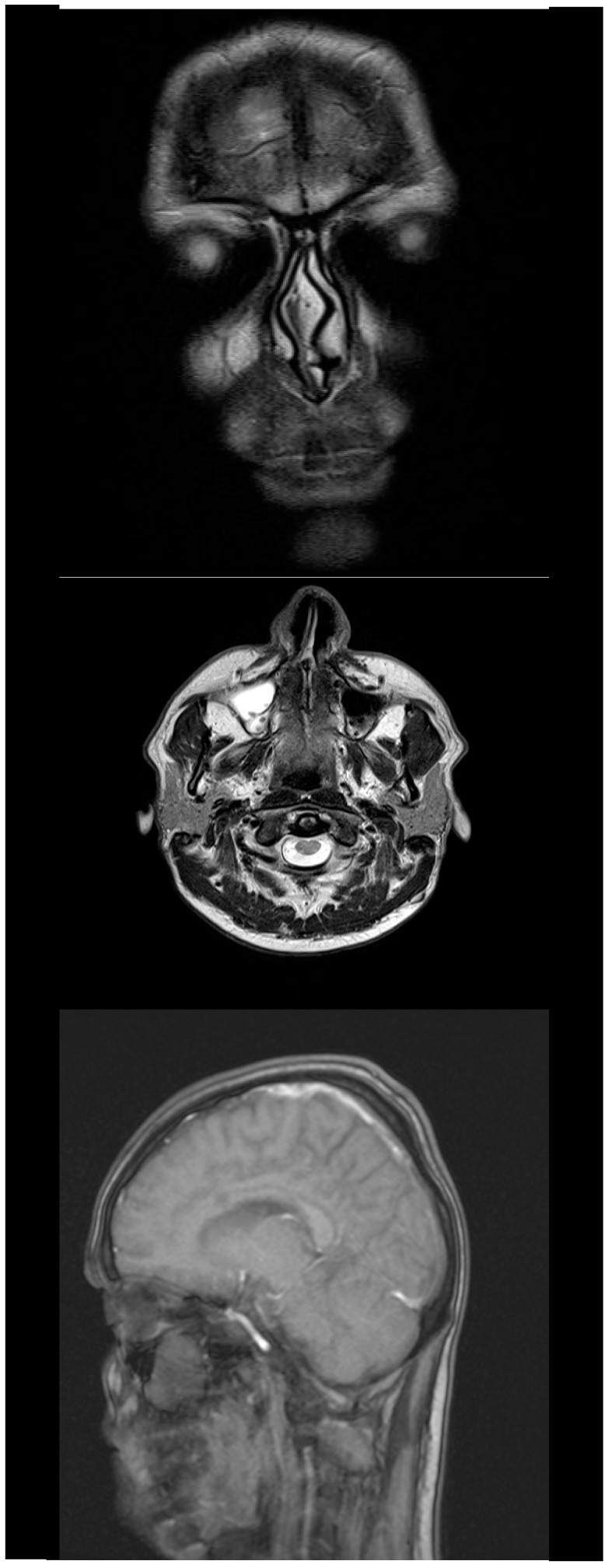
Cerebral MRI showing active lesions

**Differential diagnosis**

Although an underlying systemic disease is not often found on the initial evaluation in patients with retrobulbar neuritis, the following entities are taken into account in establishing the differential diagnosis, when neurologic signs are present: Devic’s disease, infectious diseases (Lyme Borreliosis, hepatitis B, cytomegalovirus, and herpes simplex), toxic neuropathy, and autoimmune disorders. In this case, the cerebral MRI supported the diagnosis of multiple sclerosis with ophthalmological onset.

Tests for other autoimmune disorders (pANCA, cANCA, totalANCA) and for Borreliosis have been performed and the results were negative. 

In cooperation with the neurologist, the treatment with intravenous (iv) corticosteroids (CS) was initiated: Methylprednisolone 500 mg/ day for 5 days, then 250 mg/ day for 2 days, followed by oral cortico-therapy.

At discharge, BCVA increased to 0,3 in the right eye and 0,5 in the left eye, with the persistence of a slight limitation of adduction in both eyes. 

This case was included in the National Multiple Sclerosis Treatment Program and the patient started the Betaferon treatment.

At the 3 months evaluation, BCVA increased to the values of 0,5 and 1 for the right and left eye, respectively. The fundus exam was normal and dyschromatopsia in red-green axis persisted in both eyes. The immunomodulatory treatment was continued according to the indication of the neurologist. 

## Discussions 

Most often, the term retrobulbar optic neuritis (ON) refers to the optic neuropathy associated with a demyelinating disease. Optic neuritis is often present at the onset of MS and represents a common cause of visual loss in these patients [**3**]. 

It is estimated that in 15-20% of the MS cases, the onset manifests as optic neuritis and 75% of the patients have at least one episode throughout the course of their lives [**4**]. According to Rizzo and Lessel, more than 50% of the patients diagnosed with ON would develop MS in the following 15 years [**5**].

Optic neuritis associated with MS typically presents as a monocular, sometimes painful vision loss that occurs over hours to days and lasts for a few weeks. The neuro-ophthalmologic manifestations of multiple sclerosis can be divided into two main categories: those that affect the visual sensory system and those that affect the ocular motor system [**3**]. Disturbances of visual sensory function are caused by the impairment of the optic nerve in prechiasmal, chiasmal and retrochiasmal sectors. Disturbances of ocular motility or alignment may develop during the course of MS and usually result from demyelinating lesions in the brainstem that affect supranuclear, internuclear, nuclear, or fascicular pathways [**6**].

Virtually any type of visual field loss can occur, including altitudinal, arcuate, central or cecocentral scotoma, unilateral hemianopsia or quadranopsia. Visual field defects are often found also in the contralateral eye [**7**,**8**].

Acquired dyschromatopsia, in red-green axis is also present, the color deficit often being greater than the degree of visual acuity loss [**9**].

Contrast sensitivity seems to reflect disease progression and can be a valuable prognostic marker. Recent studies have demonstrated abnormalities even in patients who had a normal vision [**10**].

OCT is a useful tool in the evaluation of the retinal nerve fiber layer (RNFL) and the ganglion cell layer thickness. OCT studies have demonstrated the thinning of RNFL in patients diagnosed with optic neuritis due to multiple sclerosis. RNFL thickness reduction reflects the axonal degeneration and atrophy of the ganglion cells. OCT findings are related both to visual impairment as well as to disease progression [**11**,**12**]. OCT is also useful for the evaluation of treatment efficacy [**13**].

Magnetic resonance imaging (MRI) showing active lesions is compulsory for the establishment of the MS diagnosis. The characteristics of the demyelinating lesions include 3 mm ovoid lesions with T2 high-signal that are mostly located in periventricular area of the white matter and radiate toward the ventricular space [**14**,**15**]. Studies showed that patients with a first episode of ON, who have normal brain MRI, seem to have a lower risk of developing MS at 15 years [**16**]. 

The case discussed in this article presented with typical retrobulbar optic neuritis signs as the initial manifestation of MS, preceding other neurological signs and symptoms.

The visual field defect registered in this patient showed a slight narrowing in the inferior-nasal quadrant of both eyes. These signs are specific to demyelinating lesions of the sensory visual pathways, although the visual function damage at that moment was minimal due to early diagnosis of the disease. Acquired dyschromatopsia, in the red-green axis, was more important than the decrease of VA at the moment of diagnosis.

A short course of intravenous corticosteroids, followed by a 2-week course of oral prednisone hastened the visual function recovery and the remission of diplopia in this young patient.

Often, the diagnosis of optic neuritis is the main factor that contributes to the decision to initiate therapy in these patients. Without prior treatment with high doses of methylprednisolone, oral prednisone alone may increase the risk for recurrent ON and should be avoided [**17**,**18**].

## Conclusions 

The cooperation between the ophthalmologist and the neurologist plays an important role for both early diagnosis and periodic clinical evaluation of MS patients. As a result, the case discussed in this article benefited from inclusion in the national MS therapy program and early initiation of therapy. Both the neurological and the visual prognosis are favorable on long term.
